# Rapid fracture healing of the greater tuberosity fragment after shoulder hemiarthroplasty in a patient with daily abaloparatide injection: A case report

**DOI:** 10.1016/j.bonr.2026.101899

**Published:** 2026-01-15

**Authors:** Itsuo Joko, Mio Uchida, Masahito Hino, Shunsuke Miyaoka, Manabu Tanaka, Kazuo Kasuga, Nobuyuki Shimojo, Shigeharu Uchiyama

**Affiliations:** a Department of Orthopaedic Surgery, Okaya City Hospital, Honcho 4-11-33, Okaya, 384-8512, Japan; b Department of Orthopaedic Surgery, Shinshu University School of Medicine, Asahi 3-1-1, Matsumoto, 390-8621, Japan; c Shimojo Clinic, Teranoguchi 2005-3, Ina, 396-0003, Japan

**Keywords:** Abaloparatide, Shoulder hemiarthroplasty, Greater tuberosity, Fracture healing, Peripheral nerve injury

## Abstract

We report a case of a 64-year-old woman who developed a 4-parts shoulder fracture-dislocation with peripheral nerve injury by the displaced fragment, during abaloparatide treatment. Following shoulder hemiarthroplasty, bone union at the greater tuberosity was achieved on postoperative day 16, with remodelling completed at 6 months postoperatively. This course was exceptionally favourable. Factors potentially contributing to the early bone union progression may include the coincidental administration of abaloparatide prior to trauma and the presence of peripheral nerve injury.

## Highlights

• Radiographic union of the greater tuberosity after hemiarthroplasty was seen at postoperative day 16.
• Daily abaloparatide had been administered for 3 months before the injury.
• Concomitant peripheral nerve injury may have influenced callus formation.
• Remodelling appeared complete by 6 months postoperatively.

## 1 Introduction

Abaloparatide increases bone mass because its anabolic effects outweigh its catabolic effects on bone resorption when administered intermittently (Chandler et al., 2019; Varela et al., 2017). Although animal studies have demonstrated that its potent anabolic effects promote fracture healing (Lanske et al., 2019; Makino et al., 2024), the clinical implications remain unclear. Here, we present a case of a patient receiving abaloparatide who sustained a four-part shoulder fracture dislocation. Following shoulder hemiarthroplasty, early bone union of the greater tuberosity was achieved.

## 2 Case

**Fig. 1:**
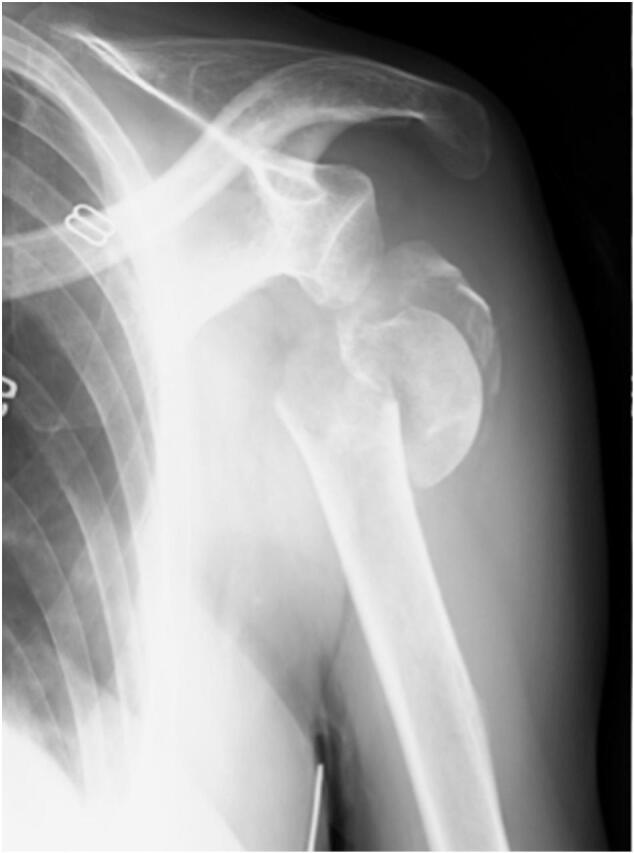
A plain radiograph of the left shoulder shows a four-part fracture dislocation, with the distal fragment displaced medially.

A 64-year-old woman fell down the stairs and sustained a fracture dislocation of the left shoulder. She was referred to our department and examined on the same day. She had previously been diagnosed with osteoporosis at a local clinic following a fragility vertebral fracture. Three months before the injury occurred, she had started taking daily abaloparatide injections (80 μg/day). Her bone mineral density (BMD) and bone turnover markers (BTM) were measured before she started taking the injections. The BMD of the lumbar spine (L2–L4) was 0.908 g/cm^3^ (T-score: −2.0) and the BMD of the right total hip was 0.61 g/cm^3^ (T-score: −2.7). Analysis of bone turnover markers demonstrated increased bone turnover, with elevated levels of both bone formation and resorption markers: type I procollagen N-propeptide (P1NP) at 127 ng/mL (reference value: 26.4–98.2 ng/mL) and tartrate-resistant acid phosphatase-5b (TRACP-5b) at 958 mU/dL (reference value: 120–420 mU/dL). Alkaline phosphatase (ALP) was measured according to the standards of the International Federation of Clinical Chemistry and Laboratory Medicine (IFCC) at 72 U/L (reference range: 38–113 U/L). She had a history of alcoholism and hypothyroidism, both of which were under control. Upon initial presentation, swelling was evident in the left shoulder. Sensory disturbance was noted on the lateral aspect of the shoulder. Deltoid muscle contraction could not be palpated. A manual muscle test of the left upper extremity revealed the following: biceps brachii 0; triceps brachii 2; brachioradialis 2; extensor carpi radialis 2; extensor digitorum communis 2; flexor pollicis longus 0; and flexor digitorum profundus 0. The radial artery was palpable and blood flow to the hand was normal. A plain radiograph revealed a four-part fracture dislocation of the left shoulder (Fig. 1). Contrast CT showed entrapment of the axillary artery by the distal fragment, but blood flow in the artery at the entrapment site was preserved.

At the first visit to our department, TRACP-5b levels were 442 mU/dL, which was lower than baseline values. However, direct comparison of bone-specific alkaline phosphatase (BAP) (15.9 μg/L, reference value:3.8–22.6 μg/L), and undercarboxylated osteocalcin (ucOC) (3.46 ng/mL, reference value:4.50 ng/mL>) was not possible due to the absence of baseline measurements. Serum 25-hydroxyvitamin D was markedly low at 7.8 ng/mL. ALP(IFCC) was slightly increased to 90 U/L (reference value:38–113 U/L). Needle EMG revealed a decreased number of motor unit action potentials, with no interference pattern in the posterior and middle fibers of the deltoid muscle. This finding was consistent with neurogenic paralysis.

Based on these findings, the diagnosis was a fracture-dislocation of the left shoulder with palsies of the axillary, median, ulnar, musculocutaneous and radial nerves, and an injury to the axillary artery.

Surgery was performed under general anesthesia on the fifth day after the injury.

**Fig. 2:**
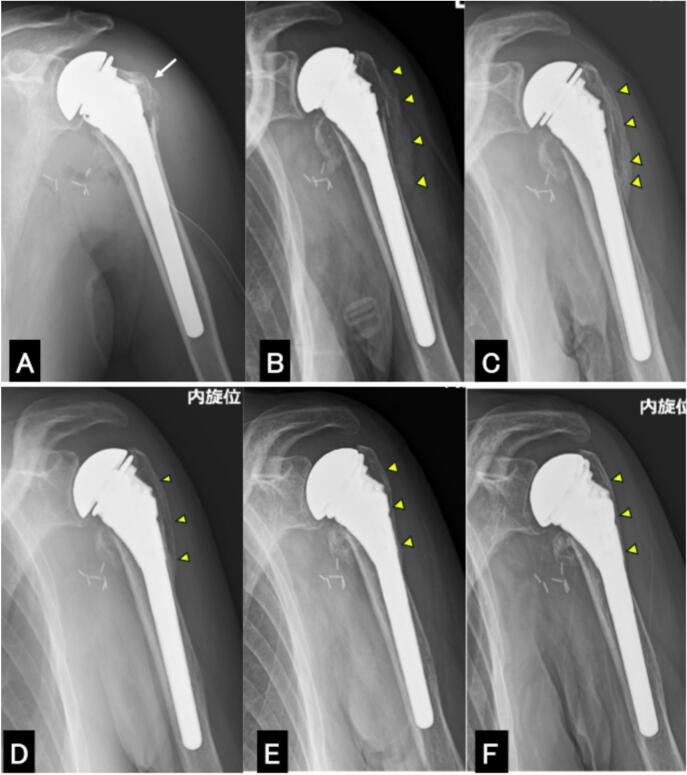
Plain anteroposterior radiographs of the left shoulder: during surgery (A), 16 days after surgery (B), 1 month after surgery (C), 3 months after surgery (D), 6 months after surgery (E), and at one year after surgery (F). A: Anatomical reduction of the greater tuberosity is achieved immediately after surgery (white arrow). B: Vigorous callus formation bridging the greater tuberosity and shaft is shown, confirming bone union (yellow arrowheads). C: The callus size has decreased and consolidation is more evident compared to 16 days after surgery, indicating a mature callus bridge over the greater tuberosity and the shaft (yellow arrowheads). D: A plain radiograph taken three months after surgery shows the beginning of remodelling of the callus (yellow arrowheads). E: A plain radiograph taken six months after surgery shows a reduction in the size of the bony bridge, suggesting progressive remodelling (yellow arrowheads). F: A plain radiograph taken one year after surgery shows no change in the size of the bony bridge, suggesting remodelling was complete by six months postoperatively (yellow arrowheads).

**Fig. 3:**
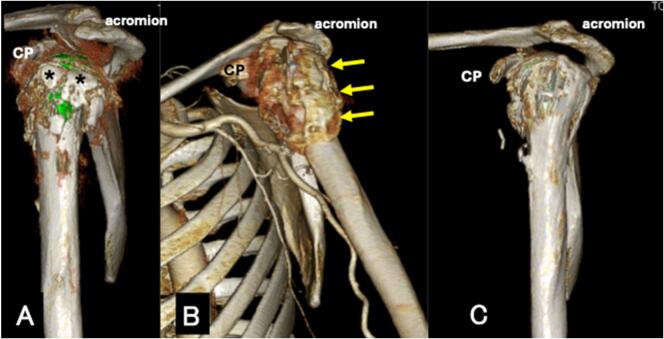
CT scan of the left shoulder immediately after surgery (A), one month (B) and one year (C) after surgery. CP: coracoid process A: Anatomical reduction of the greater tuberosity(asterisk) is seen with cancellous bone graft. B: The callus (arrow) is hardening, indicating that the bone union process is progressing favourably. C: The greater tuberosity fragment is completely healed.

First, while the patient was in the supine position, we entered via the axilla to release the entrapment of the axillary artery and identify the nerves. Only the median nerve was found to be compressed alongside the artery, and this compression was relieved. No obvious damage was noted to the ulnar or musculocutaneous nerves. Intraoperative nerve electrical stimulation elicited a slight muscle contraction in response to musculocutaneous nerve stimulation, but no response was observed in response to median or ulnar nerve stimulation. Next, shoulder hemiarthroplasty was performed via the deltopectoral approach in the beech chair position (Fig. 2A) (DepuySynthes Global Unite Standard Stem size 12, Collar Standard size 40, Epiphysis Body Porocoat size 10 + 5, Standard Humeral Head 40 × 15 mm). The stem was inserted cementless. The greater and lesser tuberosities were sutured to the shaft and head using Ethicon Nurolon suture (non-absorbable, sterile, braided surgical suture, size 2). The space between the stem and the greater tuberosity was filled with cancellous bone harvested from the excised humeral head (Fig. 3A).

Following surgery, the arm was immobilized in internal rotation using a sling. Subcutaneous abaloparatide injections were suspended for six days after surgery, after which they were resumed. No vitamin D supplements were given during the postoperative period. Plain radiograph taken on postoperative day 16 showed vigorous callus formation bridging the greater tuberosity and shaft, confirming radiographic bone union (Fig. 2B).

One month after surgery, a deltoid needle electromyography test revealed an increased number of action potentials in the anterior, middle, and posterior fibers, indicating an improvement in paralysis. Deltoid muscle contraction was confirmed by a slight palpable sensation subcutaneously. A plain radiograph taken one month after surgery showed that the callus was hardening, indicating that the bone union process was progressing favourably (Fig. 2C). A CT scan performed at one month to assess axillary artery patency also showed well-bridging callus formation between the greater tuberosity and the humeral shaft (Fig. 3B). Active and passive range of motion exercises for the left shoulder began one month after surgery. There was gradual improvement in nerve paralysis, and by three months postoperatively, active shoulder flexion had recovered to 40°, elbow flexion to 100° and finger flexion to MMT 2.

A plain radiograph taken three months after surgery showed that the callus had matured and formed a bridge between the greater tuberosity and the shaft. This indicated that remodelling of the callus had begun. (Fig. 2D). Plain radiographs (Fig. 2E) taken six months after surgery revealed a further reduction in the size of the mature callus. The BMD of the lumbar spine and total hip increased to 0.945 mg/cm^2^ (T-score: −1.5) and 0.642 mg/cm^2^ (T-score: −2.4), respectively. This suggested a positive effect of abaloparatide on BMD.

One year after surgery, active shoulder flexion was 90°, abduction was 70°, external rotation was 40° and internal rotation was limited to the L4 level. There was no pain. Regarding nerve paralysis, gradual recovery was observed with the following MMT scores: deltoid 4; biceps brachii 4; triceps brachii 4; brachioradialis 3; extensor carpi radialis 3; extensor digitorum communis 3; flexor pollicis longus 2; and flexor digitorum profundus (index and middle) 2.

Plain radiograph taken one year after surgery showed no change in the size of the bony bridge compared to that taken six months after surgery, suggesting remodelling was complete by six months postoperatively (Fig. 2F). A CT scan showed that the greater tuberosity fragment was completely healed (Fig. 3C). Abaloparatide injection was confirmed to continue one year after surgery.

## 3 Discussion

Although reverse total shoulder arthroplasty has become increasingly common for four-part proximal humerus fractures in recent years, hemiarthroplasty is also performed, particularly on younger patients. The main indications of hemiarthroplasty include a functional rotator cuff and deltoid muscle, the absence of osteoarthritis and no comminution of the greater tuberosity (Chambers et al., 2013). Although hemiarthroplasty surgery can yield favourable outcomes, bone union of the greater tuberosity significantly influences postoperative results (Holschen et al., 2018). The greater tuberosity fragment is often thin and has poor fixation strength, because fixation is achieved with sutures to the shaft. Consequently, the greater tuberosity may undergo displacement, develop nonunion, or even be resorbed before bone union is achieved. The incidence of these greater tuberosity healing complications ranges from 9% to 65%, indicating an unstable outcome (Baudi et al., 2014; Sebastiá-Forcada et al., 2014; Doursounian et al., 2019). In our patient, radiographic evidence of bone union was confirmed 16 days after surgery. Detailed reports on the process of greater tuberosity bone union are not readily found in the literature. Therefore, while definitive conclusions cannot be drawn, the observation of vigorous callus formation and cortical bridging at 16 days postoperatively, followed by well-defined lamellar bone bridging at one month postoperatively and confirmation of remodelling initiation at three months postoperatively suggests a favourable and rapid course. Remodelling completion is considered to take 6 months postoperatively, but it is known to take several months to years, making this course exceptionally favourable.

No patient-related factors that were particularly unfavorable for bone union were identified in our patient (Hernandez et al., 2012). However, severe vitamin D deficiency, which is generally considered an unfavorable factor for fracture healing, was not corrected during the postoperative period in this patient and should be recognized as a limitation of this case. One possible factor contributing to the early progression of bone union is the administration of abaloparatide. Abaloparatide is thought to exert its effects during the second phase of fracture healing, the reparative phase, which is marked by angiogenesis, cartilage formation, and development of the soft callus. Abaloparatide promotes the proliferation and differentiation of mesenchymal stem cells, chondrogenic progenitor cells and osteoblast progenitor cells. Furthermore, it enhances the maturation of chondrocytes, the production of bone matrix proteins and osteoclast formation, thereby promoting fracture healing (Lanske et al., 2019). The increase in callus formation is thought to result from the enhanced proliferation and differentiation of osteoblasts and osteoblast precursor cells (Chandler et al., 2019).

In our patient, abaloparatide was coincidentally administered for three months prior to injury. Regarding the bone-healing-promoting effect of abaloparatide administered before fracture surgery, Kataoka et al. have published a report in a preclinical study. Preoperative treatment with abaloparatide plus zoledronate accelerates femoral bone healing in rats following osteotomy. In groups receiving abaloparatide or abaloparatide plus zoledronate, starting six weeks before rat femoral osteotomy, immature callus formation was observed two weeks post-osteotomy, compared to the placebo or zoledronate-treated groups. Imaging showed that fracture healing was significantly accelerated by creating an anabolic window for increased callus formation, thereby promoting more rapid bone healing (Kataoka et al., 2024). Examining the changes in bone turnover markers in our patient before the initiation of abaloparatide treatment and at the time of injury suggests that an anabolic window, in which bone formation exceeded resorption, may have persisted for three months prior to surgery. This suggests that pretreatment could offer significant therapeutic benefits.

To date, there have only been two clinical reports on the use of abaloparatide for fracture healing. In a case of Freiberg's disease, abaloparatide was administered following dorsal closing-wedge osteotomy for a non-union, achieving bone union at five months post-treatment. The authors discuss the potential of abaloparatide to promote bone union in cases of non-union (Nelson and Gavin, 2021). A recent multidisciplinary case report described the use of abaloparatide in treating a complex periprosthetic femur fracture, which illustrates the ongoing clinical interest in using anabolic therapy alongside fracture management (Bhatti et al., 2025).

Another factor that may have promoted bone healing is the concomitant traumatic peripheral nerve injury. In this case, the pathophysiology of nerve paralysis is thought to be axonotmesis, and the paralysis gradually improved after the injury. Peripheral nerves are known to play an important role in fracture healing (Parker et al., 2024). The neuroregulation of fracture healing is a complex, multifaceted process involving the peripheral and central nervous systems, as well as numerous molecular factors. Within this process, peripheral sensory nerves are known to significantly promote healing through neuropeptide release (Parker et al., 2024). However, animal studies have demonstrated that large callus formation can occur early in fractures involving peripheral nerve transection. Aro et al. reported that, in a rat sciatic nerve transection model, although the density was low and the collagen matrix and inorganic content were reduced, a larger callus formed earlier and bridged the tibial fracture site (Aro, 1985). Similarly, Madsen et al. demonstrated increased callus formation in rat fractures following sciatic nerve resection, as evidenced by radiographic findings. However, they noted that this increased callus exhibited reduced mechanical strength, indicating a defect in callus formation (Madsen et al., 1998).

Therefore, the early and vigorous callus formation observed in this patient's greater tuberosity bone healing process on imaging may have been influenced by the effects of abaloparatide and peripheral nerve injury. However, the quality of the callus could not be assessed, and it remains unclear whether the vigorous callus formation was accompanied by adequate mechanical strength.

Abaloparatide is a parathyroid hormone-related protein (PTHrP) analogue which acts as a PTH1 receptor agonist. It shares key downstream signalling pathways with teriparatide (PTH 1–34). Therefore, existing human evidence on teriparatide and fracture healing is clinically relevant to our patient. While some case reports and case series suggest that teriparatide may enhance callus formation and promote successful fracture healing, results from clinical trials have been mixed. For example, data on proximal humerus fractures show no clear radiographic or functional benefit (Johansson, 2016). In the context of shoulder arthroplasty, teriparatide has also been reported to promote tuberosity healing following reverse total shoulder arthroplasty for acute complex proximal humeral fractures, indicating a potential biological role in tuberosity repair. The authors found clinical difference between teriparatide and no-teriparatide groups in time to achieve the initial callus (33 ± 18.3 days vs. 150 ± 42.4 days) and tuberosity consolidation (165.8 ± 70.3 days vs. 315 ± 106.1 days) in the plain radiographs (Chernchujit and Prasetia, 2018). Taken together, these observations do not prove causation, but they suggest that PTH1 receptor agonists may influence reparative processes. In our patient, the unusually rapid radiographic bridging callus formation may have been influenced by multiple factors, including pre-injury abaloparatide exposure and concomitant peripheral nerve injury.

Although greater tuberosity bone union was achieved early in our patient, shoulder functional recovery was significantly delayed. This is thought to be due to deltoid muscle dysfunction caused by axillary nerve palsy. One year postoperatively, active elevation reached 90 degrees and external rotation reached 40 degrees. Deltoid muscle strength also recovered, suggesting that further improvement in elevation is possible in the future.

## CRediT authorship contribution statement

**Itsuo Joko:** Writing – review & editing, Writing – original draft, Investigation, Data curation. **Mio Uchida:** Writing – review & editing, Investigation, Data curation. **Masahito Hino:** Writing – review & editing, Investigation, Data curation. **Shunsuke Miyaoka:** Writing – review & editing, Investigation. **Manabu Tanaka:** Writing – review & editing, Investigation, Data curation. **Kazuo Kasuga:** Writing – review & editing, Investigation. **Nobuyuki Shimojo:** Writing – review & editing, Investigation, Data curation. **Shigeharu Uchiyama:** Writing – review & editing, Supervision, Conceptualization.

## Ethical form

The patient was informed that the case data would be submitted for publication, and she gave her written consent.

## Declaration of Generative AI and AI-assisted technologies in the writing process

During the preparation of this work the author used [DeepL and ChatGPT Plus] in order to refine English. After using this tool/service, the author(s) reviewed and edited the content as needed and take(s) full responsibility for the content of the published article.

## Declaration of competing interest

The authors declare that they have no known competing financial interests or personal relationships that could have appeared to influence the work reported in this paper.

## Data Availability

Data will be made available on request.

## References

[bb0005] Aro H. (1985). Effect of nerve injury on fracture healing. Callus formation studied in the rat. Acta Orthop. Scand..

[bb0010] Baudi P., Campochiaro G., Serafini F., Gazzotti G., Matino G., Rovesta C., Catani F. (2014). Hemiarthroplasty versus reverse shoulder arthroplasty: comparative study of functional and radiological outcomes in the treatment of acute proximal humerus fracture. Musculoskelet. Surg..

[bb0015] Bhatti P., Ortiz S., Neitzke C.C., Lane J.M., Gausden E.B. (2025). Multidisciplinary approach to a transverse periprosthetic femur fracture in a short-statured patient: a case report. JBJS Case Connect..

[bb0020] Chambers L., Dines J.S., Lorich D.G., Dines D.M. (2013). Hemiarthroplasty for proximal humerus fractures. Curr. Rev. Musculoskelet. Med..

[bb0025] Chandler H., Lanske B., Varela A., Guillot M., Boyer M., Brown J., Pierce A., Ominsky M., Mitlak B., Baron R., Kostenuik P., Hattersley G. (2019). Abaloparatide, a novel osteoanabolic PTHrP analog, increases cortical and trabecular bone mass and architecture in orchiectomized rats by increasing bone formation without increasing bone resorption. Bone.

[bb0030] Chernchujit B., Prasetia R. (2018). The role of teriparatide in tuberosity healing after reverse shoulder arthroplasty in complex proximal humeral fragility fracture. J. Orthop. Surg. (Hong Kong).

[bb0035] Doursounian L., Gaillard J., Cambon-Binder A., Zbili D., Sautet A. (2019). Hemiarthroplasty for proximal humerus fractures with conservation of the whole humeral head as autograft: does it improve greater tuberosity healing?. Int. Orthop..

[bb0040] Hernandez R.K., Do T.P., Critchlow C.W., Dent R.E., Jick S.S. (2012). Patient-related risk factors for fracture healing complications in the United Kingdom General Practice Research Database. Acta Orthop..

[bb0045] Holschen M., Siemes M.K., Witt K.A., Steinbeck J. (2018). Five-year outcome after conversion of a hemiarthroplasty when used for the treatment of a proximal humeral fracture to a reverse total shoulder arthroplasty. Bone Joint J..

[bb0050] Johansson T. (2016). PTH 1-34 (teriparatide) may not improve healing in proximal humerus fractures. A randomized, controlled study of 40 patients. Acta Orthop..

[bb0055] Kataoka T., Tsubouchi Y., Takase R., Otsu T., Iwasaki T., Kataoka M., Kaku N. (2024). Preoperative abaloparatide plus zoledronate treatment accelerates femoral bone healing in rats following osteotomy. J. Orthop. Surg. (Hong Kong).

[bb0060] Lanske B., Chandler H., Pierce H., Brown J., Ominsky M., Kostenuik P., Hattersley G. (2019). Abaloparatide, a PTH receptor agonist with homology to PTHrP, enhances callus bridging and biomechanical properties in rats with femoral fracture. J. Orthop. Res..

[bb0065] Madsen J.E., Hukkanen M., Aune A.K., Basran I., Møller J.F., Polak J.M., Nordsletten L. (1998). Fracture healing and callus innervation after peripheral nerve resection in rats. Clin. Orthop. Relat. Res..

[bb0070] Makino A., Hasegawa T., Yamamoto T., Takagi H., Takahashi Y., Miyakoshi N., Amizuka N. (2024). Abaloparatide promotes bone repair of vertebral defects in ovariectomized rats by increasing bone formation. Bone.

[bb0075] Nelson J.A., Gavin K.J. (2021). The use of abaloparatide to treat nonunion following dorsal closing wedge osteotomy for Freiberg’s disease: a case report. Bone Rep..

[bb0080] Parker R.S., Nazzal M.K., Morris A.J., Fehrenbacher J.C., White F.A., Kacena M.A., Natoli R.M. (2024). Role of the neurologic system in fracture healing: an extensive review. Curr. Osteoporos. Rep..

[bb0085] Sebastiá-Forcada E., Cebrián-Gómez R., Lizaur-Utrilla A., Gil-Guillén V. (2014). Reverse shoulder arthroplasty versus hemiarthroplasty for acute proximal humeral fractures. A blinded, randomized, controlled, prospective study. J. Shoulder Elbow Surg..

[bb0090] Varela A., Chouinard L., Lesage E., Smith S.Y., Hattersley G. (2017). One year of abaloparatide, a selective activator of the PTH1 receptor, increased bone formation and bone mass in osteopenic ovariectomized rats without increasing bone resorption. J. Bone Miner. Res..

